# IV segment portal vein reconstruction in split-liver transplantation with extended right grafts

**DOI:** 10.1186/s12893-022-01761-2

**Published:** 2022-08-11

**Authors:** Dong Wang, Ning Fan, Xin Wang, Yandong Sun, Ge Guan, Jianhong Wang, Xiaodan Zhu, Yunjin Zang, Jinzhen Cai, Yuan Guo

**Affiliations:** 1 Department of Liver Disease Center, The Affiliated Hospital of Qingdao University, Qingdao, 266000 Shandong China; 2 Department of Organ Transplantation Center, The Affiliated Hospital of Qingdao University, Qingdao, 266000 Shandong China

**Keywords:** Split liver transplantation, IV segment, Ischemia, P4 reconstruction

## Abstract

**Background:**

Liver transplantation is one of the most effective treatments for end-stage liver disease. Split liver transplantation (SLT) can effectively improve the utilization efficiency of grafts. However, split liver transplantation still faces shortcomings and is not widely used in surgery. How to improve the effective transplantation volume of split liver transplantation and promote the postoperative recovery of patients has important clinical significance.

**Methods:**

In our study, the donor’s liver was split into the extended right graft and left lateral sector, and the IV segment occur ischemia. To guarantee the functional graft size, and avoid complications, we reconstructed the IV segment portal vein and left portal vein. And we analyzed the operation time, intraoperative bleeding, liver function, and postoperative complications.

**Results:**

In our research, 14 patients underwent IV segment portal vein reconstruction, and 8 patients did not undergo vascular reconstruction. We found that the ischemic area of the IV segment decreased significantly after IV segment portal vein reconstruction. We found that there was no significant difference in operation time and postoperative complications between the patients of the groups. There were significant differences in ALT on the 1st day and albumin on the 6th day after the operation.

**Conclusion:**

It indicates that IV segment reconstruction in SLT surgery can alleviate the graft ischemic and promote the recovery of liver function after the operation. And, IV segment reconstruction as a novel operating procedure may be widely used in SLT.

## Background

Liver transplantation is an effective treatment method for end-stage liver disease and liver malignant tumors [1]. With the development of surgery technology, immunosuppressive drugs, and perioperative management, the survival rate of liver transplantation has exceeded 75% in 5 years, especially in advanced liver disease. Due to the unbalanced between the number on the liver transplantation waiting list and available donor grafts. Therefore, expanding the number of grafts has important clinical significance for patients who had end-stage liver disease [2].

This severe shortage of grafts has stimulated split-liver transplantation (SLT), which was firstly introduced in the late 1980s and has had rapid development in recent years [3, 4]. SLT is based on the theory of the liver as a functional segmented organ and divided the whole liver graft into two recipients an extended right graft (ERG) given to an adult and a smaller left lateral segment to a child [5]. The emergence of SLT can effectively increase the number of liver grafts in children without reducing the number of adults [6]. SLT has greatly decreased the wait-list mortality both in pediatric and adult liver patients [7].

Up to now, SLT is widely adopted, but current studies have found that the complications and the long-term effect of SLT are not satisfactory. In contrast to whole liver transplantation, there are many technical challenges in SLT [8, 9]. For adult recipients, the small liver syndrome is the main cause of death after SLT and it is also the main difficulty to be overcome. After the whole liver graft is split, we would lose more functional graft size (FGS). In the surgery, we found that the IV segment had an ischemia region in extended right grafts (Fig. 2). On the one hand, these ischemia areas in the IV segment could decrease the volume of FGS, on the other hand, the ischemia can promote the reactive oxygen species (ROS) generation by the hepatocytes, which trigger apoptosis and necrosis in liver tissue [10]. All those can decrease the volume of FGS, what we can do for the ischemia in the extended right grafts?

With the deepening research on small liver syndrome, it has been found that FGS is an independent risk factor for a small liver syndrome which can lead to severe complications post-operation [11]. Therefore, eliminating the ischemia in the IV segment can increase the FGS which may promote the recovery of patients, and decrease the risk of infection and bleeding post-operation. And, increasing FGS as much as possible has great significance in SLT. In our study, we explored the reconstruction of the IV portal vein in SLT to eliminate the ischemia and ensure the blood supply of the IV segment. Aim to reduce the damage of FGS, promote the patient’s recovery after liver transplantation, and summarize the application value of IV segment portal vein reconstruction in split-liver transplantation with extended right grafts.

## Methods

### Study population

From January 2016 to April 2021, 22 patients underwent SLT by retrospective study, the extended right grafts for the adult patients, left lateral segment to a child patient in the Organ Transplant Center of the Affiliated Hospital of Qingdao University, and 14 patients underwent IV segment portal vein reconstruction, and 8 patients did not. And, 22 (21 adults, 1 pediatric) received an ERG. In our research, we had not analyzed the child and just analyzed the IV segment reconstruction in adults. All patients signed informed consent, and our study was approved by the Ethics Committee of the Affiliated Hospital of Qingdao University.

### Criteria for donor group

SLT donor selection criteria: BMI < 26 kg/m^2^. ICU stay is less than 5 days. The proportion of hepatic steatosis was less than 10%, and AST/ALT is less than three times the normal limit. Total bilirubin was 2 times the normal upper limit. Cold ischemia time was less than 10 h. Donor no obvious blood vessels, bile duct variation.

### Clinical data and follow-up

The age, alanine aminotransferase, aspartate aminotransferase, bilirubin, creatinine, prothrombin time, platelet, ABO blood group, weight, body mass index (BMI), donor-to-recipient weight ratio (DRWR) and other basic clinical data of the recipients before transplantation were collected. We also collected the operation time, intraoperative bleeding, blood transfusion, and other clinical data were collected. The clinical data of alanine aminotransferase, aspartate aminotransferase, bilirubin, creatinine, platelet, and prothrombin time within 9 days after operation were collected.

### Operative procedure

The liver was splited into an LLS (segments II and III) and an ERG (segments I plus IV–VIII) in vivo. The liver parenchyma, the portal vein, biliary tract, hepatic artery, and liver vein were also splited into the part of LLS and ERG. All the vessels were separated in vitro. In the IV segment portal vein reconstruction group, we also got the iliac vein from the donor to rebuild the S4 portal vein. In the operation of liver splited, the intraoperative ultrasound was used to ensure accuracy and reduce the liver and vascular damage. And when we got the donor iliac vein, the ultrasound was also used. In the process of acquiring the liver and blood vessels from the donor, we show full respect to the donor.

**Fig. 1:**
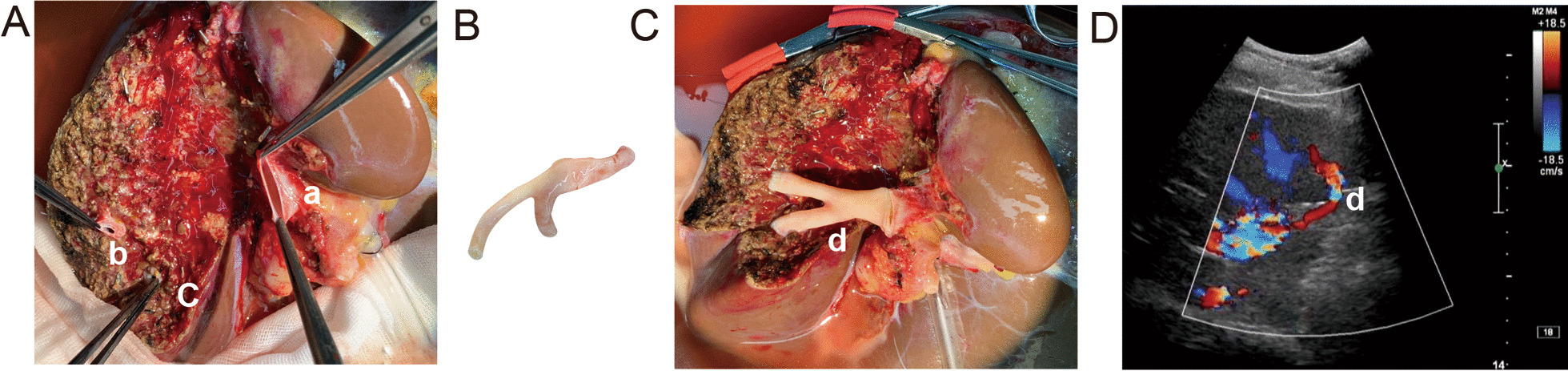
The IV segment portal vein reconstruction. **A** The extended right grafts. The ‘a stands for the left portal vein, and ‘b’ and ‘c’ mean the IV segment portal vein. **B** Iliac vein of dornor. **C** The reconstruction of IV-segment and left portal vein by the iliac vein. ‘d’ stand for the reconstruction vein. **D** The reconstruction vein by the color doppler ultrasound

The donor’s liver was placed in 4 °C UW solution. We cut off the portal vein at the root of the left branch of the portal vein, and the main portal vein was left to the right tri-lobe third lobe of the liver. Leave the middle hepatic vein to the right tri-lobe and cut off the left hepatic vein. Trim the portal vein, and ligated the small branches of the portal vein. We trimmed the common bile duct to the upper edge of the pancreas and appropriate preservation of the surrounding tissue of the common bile duct to ensure blood supply to the biliary tract. The iliac vein was also trimmed in vitro, and we reconstructed the left branch of the portal vein and the segment IV portal vein branch using the donor iliac vein. We splited the graft into the left lateral graft (segments II and III) and an extended right graft (segments I plus IV–VIII), and just as shown in Fig. 1, the IV segment portal vein was reconstructed.

And then, the common hepatic artery, biliary tract, and portal vein were separated. The second hepatic portal was anatomically analyzed, and the superior and inferior vena cava were dissociated, and then blocked superior and inferior vena cava, and the diseased liver was completely resected.

The donor’s liver was implanted in situ, and the vessel anastomosis order was the superior and inferior vena cava, inferior vena cava, and portal vein, respectively. After the portal vein anastomosis was completed, the vena cava and portal vein was opened. Recipient gastroduodenal and hepatic artery bifurcation and donor gastroduodenal and right hepatic artery bifurcation reconstruction, and open the artery. Trim the donor hepatic duct and suture with the recipient’s common bile duct. Immune induction was performed with methylprednisolone during the operation.

After the surgery, the S4 reconstruction blood flow was detected by ultrasound by blood flow velocity and construction blood anastomotic diameter. The S4 vessels were carefully examined daily for 2 weeks after surgery by a professional ultrasound doctor. For patients with an absence of blood flow in S4, we pay more attention to the liver function and the dynamic changes in patients’ recovery.

### Statistical analysis

Statistical analyses were performed using Prism software (GraphPad Prism Software, La Jolla, CA) and SPSS 21.0 (SPSS Company, Chicago, IL) for Windows. Quantitative values were analyzed by *t-*tests. Categorical variables were compared using the Chi-square test or Fisher’s exact test. P < 0.05 was considered statistically significant.

## Results

### Portal vein reconstruction can significantly eliminate ischemia in the IV segment

**Fig. 2:**
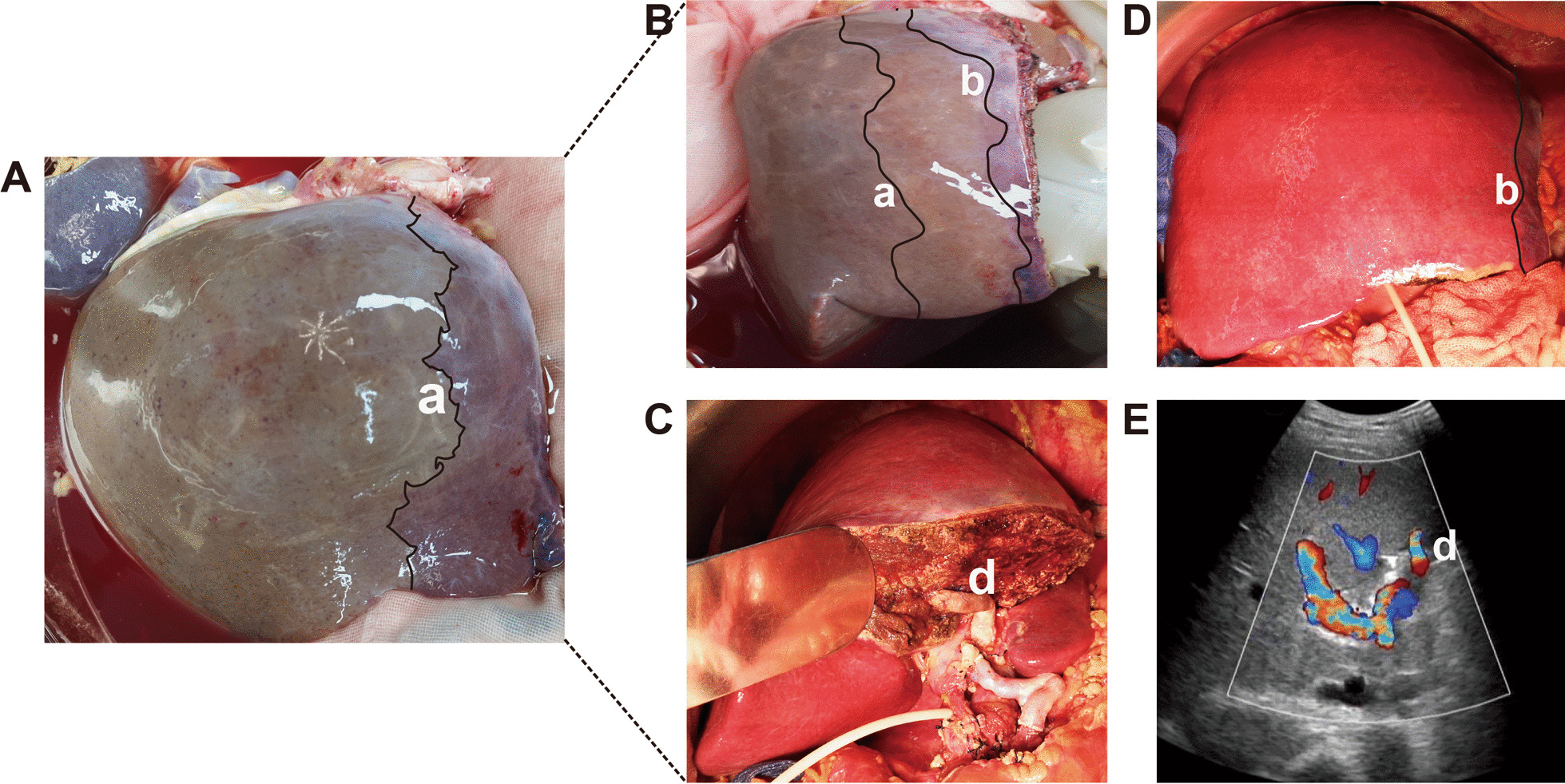
The ischemic of extended right grafts in SLT before and after IV-segment portal vein reconstruction. **A** The ischemic region in the ERG and ‘a’ mean the ischemic area. **B** The IV segment was reconstructed and we can find that the ischemic region was decreased. ‘b’ stands for the ischemic region. **C** The reconstruction vein in the extended right graft, ‘d’ means the reconstruction vein. **E** The reconstruction vein was tested by ultrasound, we can see the blood vessel patency (d)

During the transplantation surgery, we found that when the donor’s liver was split into LLS and an ERG, there was a significant ischemic area in the IV segment ERG (Fig. 2A). To reduce ischemic areas and increase the volume of functional hepatocytes, just as shown in Fig. 2-B, we used the donor iliac vein to reconstruct the left branch of the portal vein and the IV segment portal vein. We used the donor blood to reconstruct the vein between the IV segment and the left portal vein. In the surgery, we reconstructed the IV-a-segment vein, IV-b segment vein, and left portal vein. And, we reconstructed the blood between the IV-a (or IV-b) segment portal vein and the left portal vein. After vascular reconstruction, the liver ischemia region was significantly alleviated (Fig. 2-B, -D) After liver transplantation, the hepatic ischemic line was only at the surgery margin, and the blood flow in the reconstructed vessel was unobstructed (Fig. 2-E, D).

In the surgery, we found that when the liver was splited, the IV segment of ERG (segments I plus IV–VII) has areas of ischemia (Fig. 2-A). The obvious ischemic area in the IV segment may lead to the following adverse consequences. Firstly, reduce the volume of the functional liver and lead to liver failure after liver transplantation. Secondly, the ischemic in the IV segment may also become the source of abdominal cavity infection and seriously affect SLT postoperative recovery. Finally, the IV segment ischemic area may secrete more inflammatory factors, which affects the immune-inflammatory state of the body, and may interfere with the postoperative management of patients.

What we can do to the ischemic area of stage IV? In our study, we used donor veins to reconstruct the vessels in the IV segment, and after the liver transplantation, we observed that the ischemic area was significantly reduced. Therefore, it can be concluded that IV segment portal vein reconstruction can effectively reduce the volume of the ischemic liver, improve the volume of the effective liver, and finally promote the recovery of patients’ liver function. IV segment portal vein reconstruction is an innovative procedure that may be widely used in SLT.

### Clinical characteristics of enrolled patients

In the process of transplantation, we were surprised to find that the reconstruction of the IV segment portal vein could significantly improve the ischemia. Therefore, we included 21 people in this study 14 underwent IV segment reconstruction and 7 did not. All patients received an extended right part of the liver as a graft, 1 patient died of multiple organ dysfunction after transplantation, and the remaining 21 patients were successfully discharged. The average age of the patients was (45.67 ± 15.61) years, BMI was (22.53 ± 2.98) kg/m^2^, and GRWR was (2.92 ± 1.96) %. There were 12 male patients, and 9 female patients as shown in Table 1.

**Table 1:** Recipient characteristics

No	Gender	Blood type	Age (year)	Height (cm)	Weight (kg)	BMI (kg/m^2^)	GRWR (%)	IV segment portal vein reconstruction
Patient 1	Female	B	38	177	63	20.1	1.85	Yes
Patient 2	Male	O	15	170	43	14.9	2.92	No
Patient 3	Female	B	42	160	60	23.4	2.40	Yes
Patient 4	Male	O	52	168	70	24.8	1.99	Yes
Patient 5	Male	O	43	180	84	25.9	1.24	No
Patient 6	Female	O	42	163	64	24.1	2.29	No
Patient 7	Female	A	59	150	40	17.8	2.83	No
Patient 8	Male	O	39	160	62	24.2	1.25	Yes
Patient 9	Female	O	59	150	56	24.9	1.91	Yes
Patient 10	Male	O	32	172	77	26	1.76	No
Patient 11	Male	A	69	170	77	26.6	1.68	Yes
Patient 12	Male	A	25	165	59	21.7	1.88	Yes
Patient 13	Male	B	52	170	70	24.2	1.74	No
Patient 14	Male	B	45	178	63	19.9	2.21	No
Patient 15	Female	O	69	160	52	20.3	1.97	Yes
Patient 16	Female	A	63	158	55	22	1.86	Yes
Patient 17	Female	O	15	173	58	19.4	2.62	Yes
Patient 18	Male	B	63	171	74.5	24.5	1.49	Yes
Patient 19	Female	B	49	165	61	22.4	2.10	Yes
Patient 20	Male	A	37	170	65	21.5	1.89	Yes
Patient 21	Male	O	51	178	78	24.6	1.34	No

*BMI* body mass index, *GRWR* Graft Volume/Recipient Body Weight Ratio

As shown in Table 2, the glutamic-pyruvic transaminase in the vascular reconstruction group and non-vascular reconstruction group were (49.69 ± 24.49) U/L, (19 ± 7.75) U/L respectively (P < 0.05). The glutamic-oxaloacetic transaminase was (68.62 ± 32.19) U/L, (32.38 ± 29.14) U/L respectively. Total bilirubin was (145.90 ± 261.40), (36.16 ± 40.76). There was no significant difference in platelet, creatinine, length of stay, BMI, and GRWR between the vascular reconstruction group and the non-vascular reconstruction group (P > 0.05).

**Table 2:** The characteristics in the group of IV segment portal vein reconstruction

Characteristics	IV segment portal vein reconstruction		P-value
	Yes (n = 13)	No (n = 8)	
Age, years	47.69 ± 16.89	42.38 ± 13.67	0.46
BMI, kg/m^2^	22.75 ± 2.18	22.18 ± 4.13	0.68
Platelet, 10^9^/L	142.08 ± 116.66	107.75 ± 53.00	0.45
GRWR, %	1.915 ± 0.35	2.04 ± 0.63	0.57
Hospitalization time, days	46.31 ± 11.15	40.5 ± 14.33	0.31
ALT, U/L	49.69 ± 24.49	19 ± 7.75	0.003
AST, U/L	68.62 ± 32.19	32.38 ± 29.14	0.018
Bil, μmol/L	145.90 ± 261.40	36.16 ± 40.76	0.26
Serum albumin, g/L	32.72 ± 5.90	35.75 ± 7.85	0.33
PT, s	16.04 ± 4.33	16.19 ± 3.75	0.94
GGT, U/L	101.15 ± 93.65	55.38 ± 71.42	0.25
Creatinine, μmol/L	138.09 ± 245.01	70.49 ± 29.12	0.48
Gender			
Female	7	2	0.37
Male	6	6	
Blood type			
A	4	1	0.51
B	4	2	
O	5	5	
Cause of disease			
Liver failure	7	4	1
Tumor	6	4	

*BMI* body mass index, *GRWR* Graft Volume/Recipient Body Weight Ratio, *GGT* γ –glutamyltransferase, *TBil* total bilirubin; *PT* Prothrombin time, *ALT* glutamic-pyruvic transaminase, *AST* glutamic oxalacetic transaminase

### Intraoperative data analysis

We also collected the patient’s operation time, intraoperative bleeding, intraoperative blood transfusion, postoperative ICU monitoring time, and other clinical data, we found that the two groups of patients in the operation time, intraoperative bleeding, intraoperative blood transfusion, postoperative ICU monitoring had not statistically significant (P > 0.05) (Table 3). It is further proved that IV segment portal vein reconstruction does not increase the operation time and intraoperative bleeding, indicating that vascular reconstruction has high operability and safety.

**Table 3:** The operation characteristics in the two groups

Characteristics	IV segment portal vein reconstruction		P-value
	Yes (n = 13)	No (n = 8)	
The weight of the graft	1236.38 ± 159.37	1187 ± 200.37	0.56
Total OR time, min	555.63 ± 90.57	590.231 ± 113.24	0.47
Anhepatic phase, min	50.63 ± 8.28	61 ± 20.00	0.18
Hemorrhage, mL	1437.5 ± 821.04	1361.54 ± 818.07	0.84
Red blood cells transfusion volume, u	10.06 ± 6.56	10.5 ± 5.26	0.87
Plasma transfusion volume, mL	987.75 ± 534.99	1164.62 ± 651.86	0.53
ICU hospitalization time, day	6.75 ± 8.24	6.85 ± 4.04	0.97
Hospitalization time of OR, day	33.5 ± 12.35	36.54 ± 8.93	0.52

### Postoperative liver function

We collected the liver function after the operation. and had found that the alanine aminotransferase of the non-vascular reconstruction group and vascular reconstruction group on the 1st day after operation were (904.13 ± 635.23) U/L and (443.77 ± 232.17), respectively (P < 0.05) (Table 4). The serum albumin of IV reconstruction and none IV reconstruction group on the 6-day after the operation were (42.22 ± 3.22) g/L and (38.9 ± 3.49) g/L (P < 0.05). It indicated that vascular reconstruction could promote the recovery of liver function, but at the same time, there was no significant difference in bilirubin, GGT, or PLT between the two groups.

**Table 4:** The liver function after SLT in two groups

	Characteristics	IV segment portal vein reconstruction		P-value
		No (n = 8)	Yes (n = 13)	
POD-1	ALT, U/L	904.13 ± 635.23	443.77 ± 232.17	0.03
	AST, U/L	740 ± 760.79	676.77 ± 234.22	0.19
	Serum albumin, g/L	44.98 ± 8.48	39.92 ± 5.49	0.11
	PT, s	19.05 ± 2.27	20.61 ± 6.51	0.53
	Platelet, 10^9^/L	70.75 ± 27.78	81.38 ± 57.63	0.63
POD-3	ALT, U/L	491 ± 427.65	276.69 ± 177.28	0.12
	AST, U/L	110.25 ± 59.56	183.38 ± 169.79	0.26
	Serum albumin, g/L	43.09 ± 6.49	39.42 ± 4.75	0.15
	PT, s	17.08 ± 2.59	16.75 ± 3.32	0.82
	Platelet, 10^9^/L	65.13 ± 31.84	74.69 ± 68.35	0.72
POD-5	ALT, U/L	164.75 ± 149.57	143.54 ± 87.61	0.69
	AST, U/L	29.5 ± 7.56	50 ± 34.27	0.12
	Serum albumin, g/L	40.09 ± 6.35	41.17 ± 4.21	0.64
	PT, s	16.33 ± 2.42	15.05 ± 2.44	0.26
	Platelet, 10^9^/L	86.5 ± 57.5227	64.692 ± 45.3604	0.35
POD-6	ALT, U/L	109.5 ± 65.42	114.62 ± 66.85	0.87
	AST, U/L	31.63 ± 23.69	37.77 ± 28.54	0.62
	Serum albumin, g/L	38.9 ± 3.49	42.22 ± 3.22	0.04
	PT, s	15.53 ± 2.03	14.4 ± 2.27	0.27
	Platelet, 10^9^/L	96.37 ± 65.73	71 ± 46.88	0.34
POD-7	ALT, U/L	72.25 ± 50.40	95.31 ± 61.20	0.38
	AST, U/L	27.75 ± 14.59	46 ± 39.22	0.23
	Serum albumin, g/L	37.59 ± 3.97	40.33 ± 4.05	0.15
	PT, s	15.08 ± 1.85	14.95 ± 3.36	0.92
	Platelet, 10^9^/L	94.5 ± 61.63	83.23 ± 59.11	0.68

*PT* Prothrombin time, *ALT* glutamic-pyruvic transaminase, *AST* glutamic oxalacetic transaminase

## Discussion

With the successful introduction and application of the techniques of SLT waiting times and pretransplant mortality have been reduced [12]. Pro. Rudolf Pichlmayr pioneered split liver transplantation (SLT) in 1988 [13], enabling the transplantation of one donor liver into two recipients. With the development of SLT, the wait list mortality of recipients had reduced obviously [14, 15]. Split liver transplantation is an ideal method to expand the utilization of grafts and alleviate the shortage of donor livers, which can shorten the waiting time for the recipient and reduce the mortality of patients during the waiting period [16]. In recent years, with the development of surgical techniques [17], postoperative care, an immunosuppressive drug, the safety of split liver transplantation has also achieved long-term development [18, 19]. However, split liver transplantation still faces postoperative complications such as small liver syndrome, infection and biliary fistula. Therefore, it is of great clinical significance to explore how to reduce the risk of small liver syndrome after SLT surgery and improve the functional liver transplantation volume.

In the process of transplantation, the section which was splited is prone to ischemic (Fig. 1), and the ischemic part may lead to insufficient volume of effective liver transplantation and increased perioperative complications. Ensuring an adequate blood supply of grafts is important for functional transplantation. What can we do to improve marginal ischemia? In this study, we creatively reconstructed the IV segment portal vein of the graft. After reconstruction of the IV segment portal vein, we interestingly found that the part ischemic was reduced. we may conclude segment portal vein construction can reduce the risk of ischemic and increase the blood supply at the incisal margin.

The ischemia region in the IV segment occurs necrosis and may lead to serving complications. On the one hand, necrosis may further aggravate the risk of abdominal infection, biliary fistula, and even hemorrhage; on the other hand, in the immunosuppressed state after liver transplantation, the infection may be difficult to control or even lead to serious consequences, even death, due to the use of immunosuppressive drugs after surgery. We found that the IV segment reconstruction can eliminate the ischemia obviously and may reduce the incidence of complications associated with IV segment ischemia.

During the operation, we found that the ischemia of the IV segment grafts was improved after vascular reconstruction. Whether IV segment vascular reconstruction can promote postoperative recovery of patients? We further analyzed the liver function after graft vascular reconstruction. And, we found that alanine aminotransferase decreased significantly on post-operation day 1, and albumin increased significantly on the 6th day after the operation. We can conclude that vascular reconstruction can promote the recovery of liver function after an operation. IV segment grafts vascular reconstruction promoted the recovery and regeneration of liver cells at the IV segment which was ischemia. At the same time can increase the effective graft volume, prevent the occurrence of a small liver syndrome, and reduce the occurrence of postoperative liver failure.

Whether vascular reconstruction increases the operation complications. And we found that there was no increase in operation time, intraoperative bleeding, postoperative blood transfusion, postoperative hospital stays, and the risk of thoracoabdominal water after the reconstruction. All these results indicate that the reconstruction of the IV segment portal vein did not artificially increase the operation risk, and the occurrence of postoperative complications was of high operability and safety. And we can conclude that the IV segment portal vein reconstruction can relieve graft ischemia and promote liver function recovery.

In our study, although we found that the reconstruction of IV segment portal veins can promote the recovery of glutamic-pyruvic transaminase and albumin in patients, the number of cases included in this study is small, and multicenter and large samples are needed for further verification in future clinical practice. In addition, no clinical data related to liver supply were included in this study, and the effect of liver supply on liver function recovery after liver transplantation was not considered.

## Conclusions

In our study, we found that the extended right part of the liver receiving IV segment reconstruction in SLT surgery can alleviate the graft ischemic and promote the recovery of liver function.

## Data Availability

The datasets used or analyzed during the current study are available from the corresponding author on reasonable request.
